# A Randomly-Controlled Study on the Cardiac Function at the Early Stage of Return to the Plains after Short-Term Exposure to High Altitude

**DOI:** 10.1371/journal.pone.0031097

**Published:** 2012-02-17

**Authors:** Qiquan Zhou, Shengyue Yang, Yongjun Luo, Yushu Qi, Ziqiang Yan, Zifu Shi, Yong Fan

**Affiliations:** 1 Department of High Altitude Diseases, College of High Altitude Military Medicine, Third Military Medical University, Chongqing, China; 2 Key Laboratory of High Altitude Medicine of Ministry of Education, Key Laboratory of High Altitude Medicine of PLA, Chongqing, China; 3 Respiratory Medicine Center of Lanzhou Military Command, Fourth Hospital of PLA, Xining, China; 4 The 68303 Troop Hospital of People's Liberation Army, Wuwei, China; Medizinische Hochschule Hannover, Germany

## Abstract

High altitude acclimatization and adaptation mechanisms have been well clarified, however, high altitude de-adaptation mechanism remains unclear. In this study, we conducted a controlled study on cardiac functions in 96 healthy young male who rapidly entered the high altitude (3700 m) and returned to the plains (1500 m) after 50 days. Ninety eight healthy male who remained at low altitude were recruited as control group. The mean pulmonary arterial pressure (mPAP), left ventricular ejection fraction (LVEF), left ventricular fraction shortening (LVFS), cardiac function index (Tei index) were tested. Levels of serum creatine kinase isoform MB (CK-MB), lactate dehydrogenase isoenzyme-1 (LDH-1), endothelin-1 (ET-1), nitrogen oxide (NO), serum hypoxia-inducible factor-1α (HIF-1α), 8-iso-prostaglandin F_2α_ (8-iso PGF_2α_), superoxide dismutase (SOD) and malonaldehyde (MDA) were measured at an altitude of 3700 m and 1500 m respectively. The results showed that after short-term exposure to high altitude mPAP and Tei index increased significantly, while LVEF and LVFS decreased significantly. These changes were positively correlated with altitude. On the 15^th^ day after the subjects returned to low altitude, mPAP, LVEF and LVFS levels returned to the same level as those of the control subjects, but the Tei index in the returned subjects was still significantly higher than that in the control subjects (P<0.01). We also found that changes in Tei index was positively correlated with mPAP, ET-1, HIF-1α and 8-iso PGF_2α_ levels, and negatively correlated with the level of NO, LVEF, LVFS, CK-MB and LDH-1. These findings suggest that cardiac function de-adapts when returning to the plains after short-term exposure to high altitude and the function recovery takes a relatively long time.

## Introduction

High altitude deadaptation, also called “oxygen intoxication syndrome” or low altitude reaction, refers to the idiopathy involving a series of changes in functional and metabolism or even structural changes in high altitude natives or acclimatized high altitude residents when they descend to the plains. Recent studies have shown that when descending to the plains, about 50%–80% of high altitude residents or natives present a series of clinical symptoms including dizziness, palpitation, hypersomnia, malaise, chest tightening, precordial ache, arrhythmia, hypophrenia; some show abnormal readings on physiological parameters for heart, lung and blood, which continues to drop even after reaching normal plain level, i.e., overcompensated; a few individuals still present symptoms including hypoproteinemia, oligocardia, decreased cardiac function and pulmonary hypertension after two years of continuous stay in the plains; in some cases, this may last for a few years, and in the most severe cases the individuals were forced to return to high altitude. We refer to all the above pathological features as symptoms of “high altitude deadaptation reaction” or “high altitude deadaptation syndrome” [1]–[2]. Higher altitude, longer stay at high altitude and older age at return to the plains all lead to higher incidence of the disease. It is generally believed that exposure to high altitude affects cardiac function to different degrees, which increases as altitude and work intensity at high altitude increases; when individuals leave high altitude environment, these influences on heart soon disappear and the enlarged heart would quickly recover to normal. However, a recent study by our group showed that the cardiac structure of high altitude railway construction workers did not yet fully recover five years after the workers left the high altitude environment [3]. To further understand cardiac deadaptation on return to the plains after short-term exposure to high altitude, we focused on changes in pulmonary artery pressure and cardiac function on return to the plains after a 50-day exposure to high altitude, and aimed to establish characteristics of cardiac deadaptation for high altitude deadaptation syndrome.

## Results

### Changes in seroenzyme levels at different times after return to the plains from short-term exposure to high altitude

**Figure 1:**
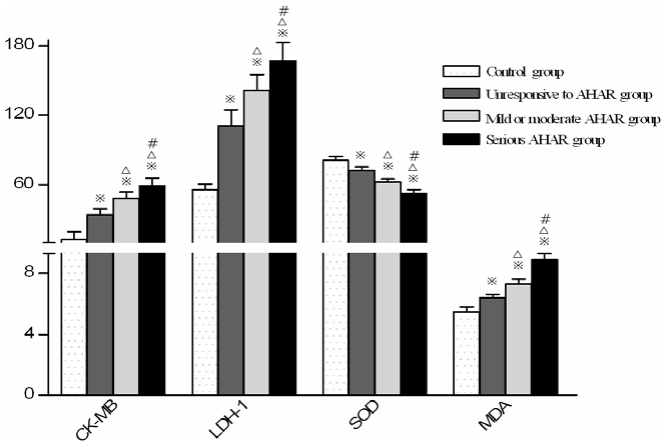
Comparison of serum myocardial enzyme and oxygen free radical levels between individuals with different degrees of high altitude reaction. (Note: 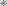 P<0.01, 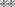
P<0.05, compared with control group; ^Δ^P<0.01 compared with unresponsive to AHAR group, ^#^ P<0.01 compared with mild and moderate AHAR group.)

**Figure 2:**
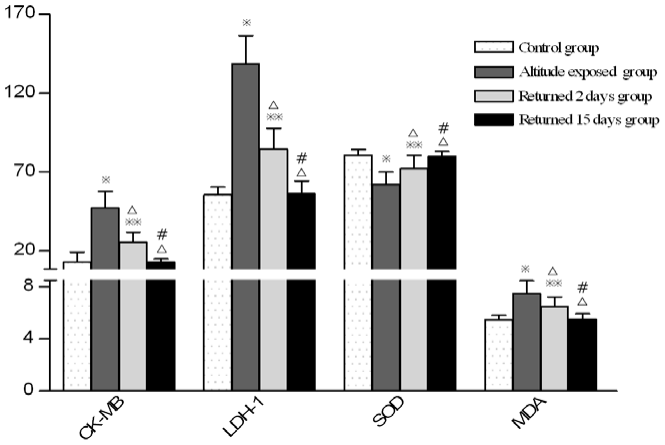
Comparison of serum myocardial enzyme and oxygen free radical levels between individuals with different times of return to the plains from high altitude. (Note: 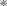 P<0.01, 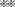
P<0.05, compared with control group; ^Δ^P<0.01 compared with altitude exposure group, ^#^ P<0.01 compared with returned 2days group.)

The serum creatine kinase isoform MB (CK-MB) concentration of test subjects after short-term exposure to high altitude was significantly higher than that of the control subjects. The mean CK-MB concentrations of the control group, test group, moderate-severe AHAR (acute high altitude reaction, also known as acute mild mountain sickness) subgroup, mild-moderate AHAR subgroup and no AHAR subgroup were shown in Table 1. The differences between the three test subgroups and the differences between each test subgroup and the control group were all significant (*P*<0.01). The differences between the CK-MB concentration of the test group at 3700 m and that on the 2^nd^, 15^th^ day of return to low altitude, and the difference between the CK-MB concentration of the test group on the 2^nd^ day of return to low altitude were significant (*P*<0.01); the CK-MB concentration of the test group on the 15^th^ day of return to low altitude was not significantly different from that of the control group (*P*>0.05, Table 1 and Figure 1–2).

**Table 1:** Test results of seroenzyme levels at different times after return to plain from short-term exposure to high altitude (U/L).

Group	Cases	50^th^ day of high altitude exposure		2^nd^ day after return to plain		15^th^ day after return to plain	
		CK-MB	LDH-1	CK-MB	LDH-1	CK-MB	LDH-1
Observation group	96	47.29±10.41	138.60±27.90	25.31±6.46	84.47±13.28	12.76±2.12	56.16±8.31
Control group	98	12.24±1.97	55.60±4.89	12.36±1.27	55.75±4.83	12.79±1.35	55.73±4.85
P value		*P*<0.01	*P*<0.01	*P*<0.01	*P*<0.01	*P*>0.05	*P*>0.05

The serum lactate dehydrogenase isoenzyme-1 (LDH-1) concentration of test subjects after short-term exposure to high altitude was significantly higher than that of the control subjects. The mean LDH-1 concentration of the control group, test group, moderate-severe AHAR subgroup, mild-moderate AHAR subgroup and no AHAR subgroup were shown in Table 1. The differences between the three test subgroups and the differences between each test subgroup and the control group were all significant (P<0.01). The differences between the LDH-1 concentration of the test group at 3700 m and that on the 2^nd^, 15^th^ day of return to low altitude, and the difference between the LDH-1 concentration of the test group on the 2^nd^ and that on the 15^th^ day of return to low altitude were all significant (P<0.01); the LDH-1 concentration of the test group on the 15^th^ day of return to low altitude was not significantly different from that of the control group (P>0.05, Table 1 and Figure 1–2).

### Changes in serum cytokine levels at different times after return to the plains from short-term exposure to high altitude

**Figure 3:**
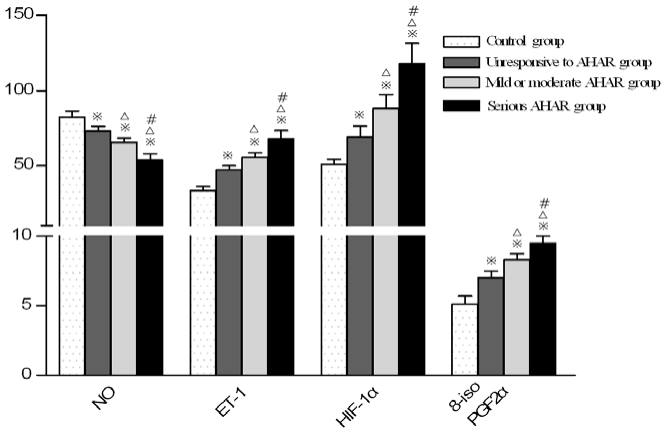
Comparison of plasma cytokine levels between individuals with different degrees of high altitude reaction. (Note: 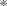; P<0.01, 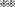
P<0.05, compared with control group; ^Δ^P<0.01 compared with unresponsive to AHAR group, ^#^ P<0.01 compared with mild and moderate AHAR group.)

**Figure 4:**
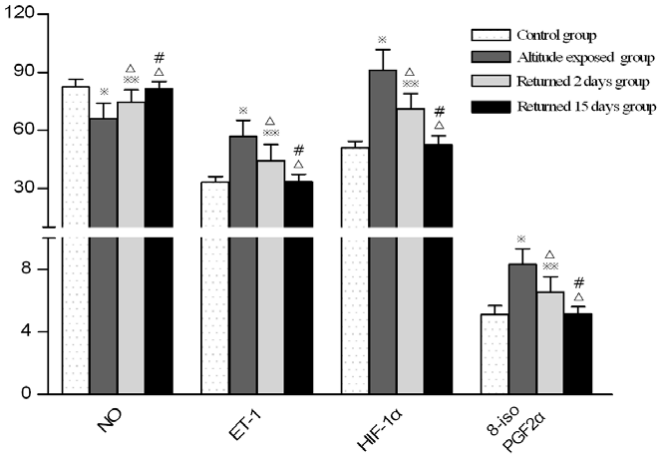
Comparison of plasma cytokine levels between individuals with different times of return to the plains from high altitude. (Note: 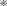; P<0.01, 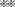
P<0.05, compared with control group; ^Δ^P<0.01 compared with altitude exposure group, ^#^ P<0.01 compared with returned 2days group.)

The serum endothelin-1 (ET-1) concentration of test subjects after short-term exposure to high altitude was significantly higher than that of the control subjects. The mean ET-1 concentration of the control group, test group, moderate-severe AHAR subgroup, mild-moderate AHAR subgroup and no AHAR subgroup were shown in Table 2. The differences between the three test subgroups and the differences between each test subgroup and the control group were all significant (P<0.01). The differences between the ET-1 concentration of the test group at 3700 m and that on the 2^nd^, 15^th^ day of return to low altitude, and the difference between the ET-1 concentration of the test group on the 2^nd^ and that on the 15^th^ day of return to low altitude were all significant (P<0.01); the ET-1 concentration of the test group on the 15^th^ day of return to low altitude was not significantly different from that of the control group (P>0.05,Table 2 and Figure 3–4).

**Table 2:** Test results of serum diastolic and systolic factor levels and pulmonary artery pressure at different times after return to plain from short-term exposure to high altitude.

Group	Cases	50^th^ day of high altitude exposure			2^nd^ day after return to plain			15^th^ day after return to plain		
		ET-1 (ng/L)	NO (µmol/L)	mPAP (mmHg)	ET-1 (ng/L)	NO (µmol/L)	mPAP (mmHg)	ET-1 (ng/L)	NO (µmol/L)	mPAP (mmHg)
Observation group	96	56.69±8.51	66.04±7.91	29.08±4.22	44.25±8.51	74.51±6.55	23.05±3.18	33.46±3.68	81.50±3.61	18.96±1.75
Control group	98	33.27±2.86	82.59±4.02	18.50±1.31	34.04±1.88	82.81±3.36	18.29±1.07	32.88±1.69	83.76±2.04	18.11±0.94
P value		*P*<0.01	*P*<0.01	*P*<0.01	*P*<0.01	*P*<0.01	*P*<0.01	*P*>0.05	*P*>0.05	*P*>0.05

The serum nitrogen oxide (NO) concentration of test subjects after short-term exposure to high altitude was significantly lower than that of the control subjects. The mean NO concentration of the control group, test group, moderate-severe AHAR subgroup, mild-moderate AHAR subgroup and no AHAR subgroup were shown in Table 2. The differences between the three test subgroups and the differences between each test subgroup and the control group were all significant (P<0.01). The differences between the NO concentration of the test group at 3700 m and that on the 2^nd^, 15^th^ day of return to low altitude, and the difference between the NO concentration of the test group on the 2^nd^ and that on the 15^th^ day of return to low altitude were all significant (P<0.01); the NO concentration of the test group on the 15^th^ day of return was not significantly different from that of the control group (P>0.05, Table 2 and Figure 3–4).

The serum hypoxia-inducible factor-1α (HIF-1α) concentration of test subjects after short-term exposure to high altitude was significantly lower than that of the control subjects. The mean HIF-1α concentration of the control group, test group, moderate-severe AHAR subgroup, mild-moderate AHAR subgroup and no AHAR subgroup were shown in Table 3. The differences between the three test subgroups and the differences between each test subgroup and the control group were all significant (P<0.01). The differences between the HIF-1α concentration of the test group at 3700 m and that on the 2^nd^, 15^th^ day of return to low altitude, and the difference between the HIF-1α concentration of the test group on the 2^nd^ and that on the 15^th^ day of return to low altitude were all significant (P<0.01); the HIF-1α concentration of the test group on the 15^th^ day of return to low altitude was not significantly different from that of the control group (P>0.05, Table 3 and Figure 3–4).

**Table 3:** Test results of serum cytokine HIF-1α and 8-iso-PGF_2_α levels at different times after return to plain from short-term exposure to high altitude.

Group	Cases	50^th^ day of high altitude exposure		2^nd^ day after return to plain		15^th^ day after return to plain	
		HIF-1α (pg/L)	8-iso-PGF_2_α (µg/L)	HIF-1α (pg/L)	8-iso-PGF_2_α (µg/L)	HIF-1α (pg/L)	8-iso-PGF_2_α (µg/L)
Observation group	96	91.16±20.58	8.33±1.02	70.99±8.22	6.54±1.01	52.31±4.92	5.16±0.47
Control group	50	50.57±3.27	5.07±0.32	52.58±2.34	5.11±0.28	53.65±1.86	5.30±0.21
P value		*P*<0.01	*P*<0.01	*P*<0.01	*P*<0.01	*P*>0.05	*P*>0.05

The serum 8-iso-prostaglandin F_2α_ (8-iso-PGF2α) concentration of test subjects after short-term exposure to high altitude was significantly lower than that of the control subjects. The mean 8-iso-PGF2α concentration of the control group, test group, moderate-severe AHAR subgroup, mild-moderate AHAR subgroup and no AHAR subgroup were shown in Table 3. The differences between the three test subgroups and the differences between each test subgroup and the control group were all significant (P<0.01). The differences between the 8-iso-PGF2α concentration of the test group at 3700 m and that on the 2^nd^, 15^th^ day of return to low altitude, and the difference between the 8-iso-PGF2α concentration of the test group on the 2^nd^ and that on the 15^th^ day of return to low altitude were all significant (P<0.01); the 8-iso-PGF2α concentration of the test group on the 15^th^ day of return to low altitude was not significantly different from that of the control group (P>0.05. Table 3 and Figure 3–4).

### Changes in blood oxygen free radical levels at different times after return to the plains from short-term exposure to high altitude

The serum superoxide dismutase (SOD) concentration of test subjects after short-term exposure to high altitude was significantly lower than that of the control subjects. The mean SOD concentration of the control group, test group, moderate-severe AHAR subgroup, mild-moderate AHAR subgroup and no AHAR subgroup were shown in Table 4. The differences between the three test subgroups and the differences between each test subgroup and the control group were all significant (P<0.01). The differences between the SOD concentration of the test group at 3700 m and that on the 2^nd^, 15^th^ day of return to low altitude, and the difference between the SOD concentration of the test group on the 2^nd^ and that on the 15^th^ day of return to low altitude were all significant (P<0.01); the SOD concentration of the test group on the 15^th^ day of return to low altitude was not significantly different from that of the control group (P>0.05, Table 4 and Figure 1–2).

**Table 4:** Test results of serum oxygen free radical SOD and MDA levels at different times after return to plain from short-term exposure to high altitude.

Group	Cases	50^th^ day of high altitude exposure		2^nd^ day after return to plain		15^th^ day after return to plain	
		SOD (nu/mL)	MDA (µmol/L)	SOD (nu/mL)	MDA (µmol/L)	SOD (nu/mL)	MDA (µmol/L)
Observation group	96	62.15±7.80	7.49±0.97	72.10±8.66	6.47±0.75	79.82±3.15	5.51±0.42
Control group	98	81.03±3.53	5.49±0.34	81.64±2.41	5.44±0.22	81.57±2.99	5.48±0.24
P value		*P*<0.01	*P*<0.01	*P*<0.01	*P*<0.01	*P*>0.05	*P*>0.05

The serum malonaldehyde (MDA) concentration of test subjects after short-term exposure to high altitude was significantly higher than that of the control subjects. The mean MDA concentration of the control group, test group, moderate-severe AHAR subgroup, mild-moderate AHAR subgroup and no AHAR subgroup were shown in Table 4. The differences between the three test subgroups and the differences between each test subgroup and the control group were all significant (P<0.01). The differences between the MDA concentration of the test group at 3700 m and that on the 2^nd^, 15^th^ day of return to low altitude, and the difference between the MDA concentration of the test group on the 2^nd^ and that on the 15^th^ day of return to low altitude were all significant (P<0.01); the MDA concentration of the test group on the 15^th^ day of return to low altitude was not significantly different from that of the control group (P>0.05, Table 4 and Figure 1–2).

### Changes in cardiac function at different times after return to the plains from short-term exposure to high altitude

**Figure 5:**
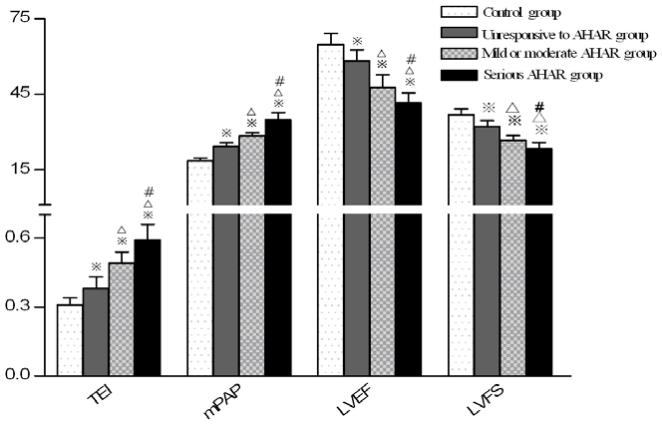
Comparison of cardiac function and pulmonary artery pressure between individuals with different degrees of high altitude reaction. (Note: 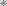; P<0.01, 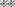
P<0.05, compared with control group; ^Δ^P<0.01 compared with unresponsive to AHAR group, ^#^ P<0.01 compared with mild and moderate AHAR group.)

**Figure 6:**
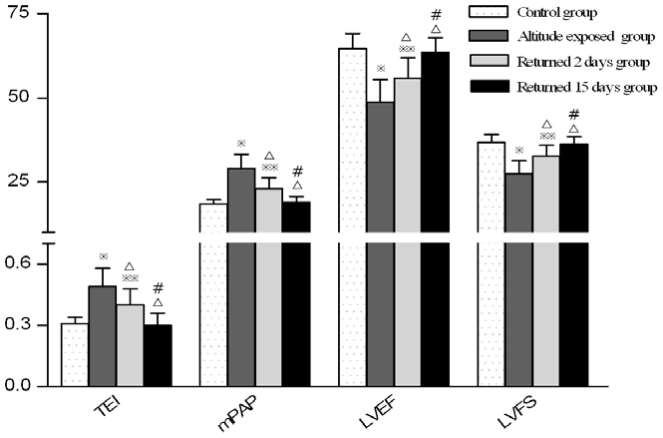
Comparison of cardiac function and pulmonary artery pressure between individuals with different times of return to the plains from high altitude. (Note: 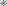; P<0.01, 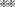
P<0.05, compared with control group; ^Δ^P<0.01 compared with altitude exposure group, ^#^ P<0.01 compared with returned 2days group.)

The cardiac function index (Tei index) of test subjects after short-term exposure to high altitude was significantly higher than that of the control subjects. The mean Tei index for the left and right ventricle of the control group, test group, moderate-severe AHAR subgroup, mild-moderate AHAR subgroup and no AHAR subgroup were shown in Table 5. The differences between the three test subgroups and the differences between each test subgroup and the control group were all significant (P<0.01). The differences between the Tei index of the test group at 3700 m and that on the 2^nd^, 15^th^ day of return to low altitude, and the difference between the Tei index of the test group on the 2^nd^ and that on the 15^th^ day of return to low altitude were all significant (P<0.01); the Tei index of the test group on the 15^th^ day of return to low altitude was still significantly different from that of the control group (P<0.05, see Table 5, Table 6 and Figure 5–6).

**Table 5:** Test results of right cardiac function at different times after return to plain from short-term exposure to high altitude.

Group	Cases	50^th^ day of high altitude exposure			2^nd^ day after return to plain			15^th^ day after return to plain		
		Tei Index	RVEF (%)	RVFS (%)	Tei Index	RVEF (%)	RVFS (%)	Tei Index	RVEF (%)	RVFS (%)
Observation group	96	0.56±0.16	45.73±5.85	25.24±3.23	0.49±0.16	46.67±5.77	27.76±3.84	0.47±0.16	61.35±4.75	34.57±3.29
Control group	98	0.33±0.03	56.85±3.78	33.73±3.58	0.32±0.02	56.95±3.38	34.23±3.18	0.34±0.03	57.27±3.35	34.89±3.27
P value		*P*<0.01	*P*<0.01	*P*<0.01	*P*<0.01	*P*<0.01	*P*<0.01	*P*<0.01	*P*>0.05	*P*>0.05

**Table 6:** Test results of left cardiac function at different times after return to plain from short-term exposure to high altitude.

Group	Cases	50^th^ day of high altitude exposure			2^nd^ day after return to plain			15^th^ day after return to plain		
		Tei Index	LVEF (%)	LVFS (%)	Tei Index	LVEF (%)	LVFS (%)	Tei Index	LVEF (%)	LVFS (%)
Observation group	96	0.49±0.09	48.71±6.81	27.42±3.93	0.40±0.08	55.87±6.07	32.76±3.14	0.40±0.08	63.50±4.45	36.27±2.19
Control group	98	0.31±0.03	64.83±4.37	36.69±2.39	0.32±0.02	64.44±3.53	36.59±2.25	0.32±0.02	64.36±3.09	36.42±1.91
P value		*P*<0.01	*P*<0.01	*P*<0.01	*P*<0.01	*P*<0.01	*P*<0.05	*P*<0.05	*P*>0.05	*P*>0.05

The left ventricular ejection fraction (LVEF) of test subjects after short-term exposure to high altitude was significantly lower than that of the control subjects. The mean LVEF and RVEF of the control group, test group, moderate-severe AHAR subgroup mild-moderate AHAR subgroup and no AHAR subgroup were summarized in Table 5 and 6. The differences between the three test subgroups and the differences between each test subgroup and the control group were all significant (P<0.01). The differences between the LVEF of the test group at 3700 m and that on the 2^nd^, 15^th^ day of return to low altitude, and the difference between the LVEF of the test group on the 2^nd^ and that on the 15^th^ day of return to low altitude were all significant (P<0.01); the LVEF of the test group on the 15^th^ day of return to low altitude was not significantly different from that of the control group (P>0.05, Table 5, Table 6 and Figure 5–6).

The left ventricular fraction shortening (LVFS) of test subjects after short-term exposure to high altitude was significantly lower than that of the control subjects. The mean LVFS and RVFS of the control group, test group, moderate-severe AHAR subgroup, mild-moderate AHAR subgroup and no AHAR subgroup were summarized in Table 5 and 6. The differences between the three test subgroups and the differences between each test subgroup and the control group were all significant (P<0.01). The differences between the LVFS of the test group at 3700 m and that on the 2^nd^, 15^th^ day of return to low altitude, and the difference between the LVFS of the test group on the 2^nd^ and that on the 15^th^ day of return to low altitude were all significant (P<0.01); the LVFS of the test group on the 15^th^ day of return to low altitude was not significantly different from that of the control group (P>0.05, Table 5, Table 6 and Figure 5–6).

The mean pulmonary arterial pressure (mPAP) of test subjects after short-term exposure to high altitude was significantly higher than that of the control subjects. The mean mPAP of the control group, test group, moderate-severe AHAR subgroup, mild-moderate AHAR subgroup and no AHAR subgroup were summarized in Table 2. The differences between the three test subgroups and the differences between each test subgroup and the control group were all significant (P<0.01). On the 2nd and that on the 15th day after return to the plains (1500 m), the mean mPAPs of the test group were 23.05±3.18 mmHg and 18.96±1.75 mmHg, respectively. The differences between the mPAP of the test group at 3700 m and that on the 2^nd^, 15^th^ day of return to low altitude, and the difference between the mPAP of the test group on the 2^nd^ and that on the 15^th^ day of return to low altitude were all significant (P<0.01); the mPAP of the test group on the 15^th^ day of return to low altitude was not significantly different from that of the control group (P>0.05, Table 2 and Figure 5–6).

## Discussion

There has been no previous report on the cardiac deadaptation during the high altitude deadaptation process and, there has no been study on the cardiac function deadaptation at return to the plains after short-term high altitude exposure. Heath et al. proposed that as high altitude residents descended to the plains, the abnormal environmental stimulation was removed and these people were no longer subject to health threat [4]–[6]. However, Vogel reported that when high altitude natives (43∼50 years old) entered sea level, the stroke index (SI) of their hearts significantly increased, heart rate significantly decreased, whereas the cardiac index (CI) remained unchanged [7]–[9]. Tei index is a new index evaluating global cardiac function using ultrasonic cardiogram, a.k.a. ventricular myocardial performance index (VMPI), first proposed by Japanese scholar Tei et al. [9] and adopted in clinical application. Tei index is a ratio of the sum of ventricular isovolumic contraction (ICT) and, isovolumic relaxation (IRT) and the ventricular ejection time (ET). The index reflects the global cardiac systolic and diastolic function. Both ICT extending, ET shortening during systolic dysfunction and extending IRT, ET shortening during diastolic dysfunction can cause an increase in Tei index. As Tei index evaluates the global cardiac function of the left ventricle, and is easy to measure and repeat, not influenced by factors including age, heart rate, geometric shape of the ventricle, ventricle systolic and diastolic pressures, it is a more scientific way to measure cardiac function than evaluating the systolic and diastolic functions of the heart alone [10], The ventricular ejection fraction (EF) reflects only the ventricular systolic function under the normal EF circumstances, while Tei index reflects the degree of ventricular diastolic dysfunction. It is more accurate to use left ventricular diastolic function as a sensitive indicator [11], which has been widely used in clinical applications. In recent years some have also calculated Tei index from right ventricular isovolumic contraction time, right ventricular isovolumic relaxation time and right ventricle ejection time to evaluate the systolic and diastolic functions of the right ventricle [12]–[13]. In this study, we found that rapid exposure to high altitude of the plains residents could lead to an increase in pulmonary artery pressure and a decrease in left ventricle function, even is the high altitude exposure was only short term; these changes were positively correlated with altitude and degree of altitude stress, i.e. higher altitude and worse altitude reaction led to more severely impaired left ventricle function. We also found the LVEF and LVFS decreased significantly. On the 15^th^ day after return to low altitude, the test subjects' LVEF and LVFS had recovered to the level of control subjects; however, the Tei index of the left and right ventricle had not, indicating a low global cardiac function at that time point. Our results suggest that cardiac deadaptation process exists in population returning to the plains after short-term exposure to high altitude; in particular, the recovery of the left and right ventricle function is slow and it may still be in the deadaptation condition 15 days after return to low altitude, which needs to be attended to clinically.

Many factors may influence the systolic and diastolic function of the right ventricle, and the pulmonary artery pressure is the most important one. It is well known that alveolar hypoxia is a direct trigger of pulmonary vessel contraction.In this study, we found that exposure to high altitude could quickly increase the vascular contracting factors and decrease the vascular relaxing factors in the plasma, after subjects return to the plains from high altitude, vascular contracting factor ET-1 release gradually decreases and vascular diastolic factor NO release gradually increases, both recover to normal level as the in the control subjects 15 days after the return to low altitude. ET-1 is the most potent internal vascular contracting factor currently known and lungs are the most important organ for the function and metabolism of ET-1 [14]. Under hypoxia the endothelial cells in the lung tissue are damaged and ET-1 gene expression is up-regulated; as the serum ET-1 concentration increases, ET-1 combines ETAR on the pulmonary vascular smooth muscles and causes opening of Ca^2+^ channels and increase in intracellular Ca^2+^ concentration, which leads to contraction of pulmonary vessels and increase in pulmonary artery pressure [15]. Selective ET-1-A antagonist can inhibit hypoxic pulmonary vessel contraction, inhibit or decrease pulmonary hypertension under hypoxia [16], through which hypoxia can regulate ET-1 gene expression via inducing HIF-1α and ET-1 levels were linearly correlated with pulmonary artery pressure. After the subjects returned to low altitude, HIF-1α and ET-1 release gradually decreased, and recovered to normal control levels in 15 days after return to low altitude. HIF -1α is an important protein regulatory factor produced by the body under hypoxia, which can regulates many target genes [17]–[18], including ET-1 and NO. Our study found that HIF -1α level is positively correlated with mPAP significantly. Therefore, changes in HIF-1α level caused by hypoxia may play an important role in the pathogenesis of hypoxic pulmonary hypertension [19]. 8-iso-PGF_2_α is a recently discovered biologically active prostaglandin-like substance. Romero et al. [20] found that 8-iso-PGF2α had strong effect in vessel contraction and antinatriuresis; it was involved in generation of high blood pressure even when Ang II level was normal. In addition, the 8-iso-PGF2α concentration in the body fluid is very stable, hence it has been considered the most ideal biochemical index for evaluating free radical oxidation intensity in living bodies [21]–[22]. Our study found that 8-iso-PGF2α level is positively correlated with mPAP significantly. Therefore hypoxia induced serum 8-iso- PGF2α level increase also plays an important role in the generation of hypoxic pulmonary hypertension. Increase in pulmonary artery pressure adds to the load on right ventricle; decrease in pulmonary artery pressure alleviates the pressure load on right ventricle and facilitates the recovery of right ventricle. Therefore, 8-iso-PGF_2_α level on right ventricular function has a direct influence.

Our results showed that exposure to high altitude led to myocardial damage; the myocardial enzyme activities increased. Upon return to the plains from high altitude, myocardial enzyme CK-MB and LDH-1 activities dropped quickly, and returned to normal control level in 15 days. Upon return to the plains from high altitude, LVEF and LVFS increased quickly, and recovered to normal control level in 15 days after return to low altitude. However, on the 15^th^ day after return, the Tei index that indicates cardiac function still remained high. In particular, acute hypoxia could cause cardiomyocyte membrane damage, leading to decrease in membrane fluidity and increase in membrane permeability [23], which led to outflow of CK-MB and LDH-1 and further aggravated myocardial damages. In addition, hypoxia could also lead to increase in generation of oxygen free radicals, which could further impair cardiac muscles. It is known that oxygen free radicals can lead to oxidation of unsaturated fatty acid in the cell membrane, inactivation of intracellular enzymes and cell membrane rupture, causing severe damages to tissues and cells [24]. Our study also found that exposure to high altitude could lead to increase in serum MDA level and decrease in SOD level; the magnitude of the changes paralleled the degree of high altitude reaction: more severe high altitude reaction led to more increase of MDA level. When subjects returned to low altitude, the MDA activity decreased and SOD level increased, both having recovered to normal control level on the 15^th^ days after the return to low altitude. It is thus evident that after return to the plains, the oxidative damage is alleviated and the antioxidative ability is increased, which leads to more removal of oxygen free radicals in the organism and restored oxidation-antioxidation balance.

In conclusion, hypoxia at high altitude can lead to increase in pulmonary artery pressure and decrease in global cardiac function at rapid exposure high altitude, which involves disturbed balance of plasma systolic and diastolic factor release and damage to the cardiac muscles. Damages to the cardiac structure and function caused by short-term rapid exposure to high altitude are reversible. When individuals return to the plains, the myocardial damages can quickly recover and the cardiac function quickly restores to normal control level in about half a month; however, full recovery of global ventricle function takes longer time. During the recovery period, one should protect cardiac muscles, avoid overtiredness and infection, in order to prevent aggravating myocardial damages which may cause high altitude deadaptation syndrome [25].

## Materials and Methods

### Subjects

Ninety six young males who quickly entered high altitude area (3700 m) from low altitude area (1500 m) and were engaged in heavy labor after “4.14” Yushu earthquake in Qinghai province were selected using random number table. All subjects gave their informed consent in writing. All test subjects were male, aging 18–35 years old with a mean of 21.8±3.6 years old. All subjects were immediately engaged in heavy labors including rescue work, loading and unloading goods and materials, clearing seismic ruins for 8∼10 hours per day. On the 50^th^ day completing their heavy labor duty at high altitude, the 2^nd^ and 15^th^ day after a 48-hour by bus return to proper station (1500 m) respectively, the subjects' mean pulmonary arterial pressure (mPAP), left ventricular ejection fraction (LVEF), left ventricular fraction shortening (LVFS), cardiac function index (Tei index), levels of serum creatine kinase isoform MB (CK-MB), lactate dehydrogenase isoenzyme-1 (LDH-1), endothelin-1 (ET-1), nitrogen oxide (NO), serum hypoxia-inducible factor-1α (HIF-1α), 8-iso-prostaglandin F_2α_ (8-iso PGF_2α_), superoxide dismutase (SOD) and malonaldehyde (MDA) were measured. Ninety eight young males from the same unit and not exposed to high altitude were used as control subjects. The age of the control subject ranged from 19 to 32 years old with a mean of 22.9±3.5 years old. This study was approved by the medical ethical committee of the Third Military Medical University.

### Acute high altitude reaction (AHAR) symptom scale assessment and scale division

On each day of the subjects' 50-day stay at high altitude, a medical doctor was responsible for examining and recording their AHAR symptoms, which were scaled and divided into groups according to Chinese AHAR semiotic assessment method: a total score of 1∼4 was normal, 5∼10 mild AHAR, 11∼15 moderate AHAR and above 16 severe AHAR [26]. Based on the scale results, 71 out of 96 (74.0%) test subjects showed AHAR, including 24 cases of severe AHAR (group A) and 47 cases of moderate AHAR (group B). There were 25 (26.0%) test subjects who did not show AHAR.

### Measurement of serum ET-1 and NO

Fasting venous blood (4 ml) was collected to anticoagulant tube containing 0.2 ml 0.2% EDTA, centrifuged at 3000 r/min 4°C (centrifugal radius 15 cm) for 10 min to separate plasma, and stored in −30°C refrigerator for testing. Radioimmunoassay method was applied to measure ET-1 level (kit provided by Beijing Dongya Immunology Research Institute); nitric acid reduction method was applied to measure NO level (kit purchased from Shenzhen Jingmei Bioengineering Inc.). All operations were performed according to instructions in the kits.

### Measurement of 8-iso PGF_2α_, SOD and MDA

Fasting venous blood (5 ml) was collected (with EDTA), centrifuged at 3000 r/min for 10 min at 4°C to separate plasma, and stored in −80°C previous testing. Enzyme liked immunosorbent assay (ELISA) was used to measure 8-iso PGF_2α_ concentration (kit purchased from ADL company, USA); operation was performed strictly according to kit instruction; optical density at 450 nm was determined with ELISA reader (Bio-Rad 3550). Chemical colorimetric analysis was performed to measure SOD and MDA levels (kit produced by Nanjing Jiancheng Bioengineering Research Institute).

### Measurement of serum HIF-1α level

Double antibody sandwich ELISA (DAS-ELISA) was used (kit purchased from Beijing Zhongshan Jinqiao Bioengineering Inc.); operation was performed strictly according to kit instruction; optical density at 450 nm was determined with ELISA reader (Bio-Rad 3550); standard curve was plotted and serum HIF-1α, VEGF level calculated.

### Measurement of myocardial enzymes

Morning fasting venous blood (3 ml) was collected (with EDTA), centrifuged at 3500 r/min for 10 min at 4°C to separate plasma, and stored in −80°C before testing. Performance rate method was applied to measure CK-MB and LDH-1 concentrations (kit purchased from Lanzhou Beiken Biology Inc.); operation was performed strictly according to kit instruction; fully automated biochemistry analyzer (BS-400) was used for reading.

### Measurement of mPAP

Color Doppler echocardiography instrument (Kangbo RT 6800) was used to measure the right ventricular pre-ejection period (RVPEP) and pulmonary artery acceleration time (AT) from pulmonary artery blood flow spectra; RVPEP refers to the time interval between the onset of Q wave in ECG and the onset of pulmonary artery systolic blood flow spectra, whereas AT refers to the time interval between the onset and the peak of pulmonary artery systolic blood flow spectra. mPAP (mmHg) = 42.1(RVPEP/AT)−15.7 [27].

### Measurement of cardiac function

Color Doppler ultrasound system (GE LOGIQ-3) was used to measure Tei index, LVEF and LVFS. The subjects took left lateral position after 1-hour rest, and the apical four-chamber and five-chamber views were obtained. (1) Pulsed Doppler sample volume was placed at mitral valve and aortic valve to record blood flow spectra when heart rate was in steady-state; the time interval between the end of mitral valve blood flow A peak and the beginning of the E peak in the next cardiac cycle (line a) and the aortic ejection time (line b) were measured. Tei index was calculated with formula Tei Index = (a−b/b) [9]. (2) Simpson's method was applied to calculate the subjects' LVEF and LVFS [28]–[29]. Right ventricular systolic and diastolic function were evaluated by measuring the spectrum of tricuspid annular wall motion according to the literature [30]–[31].

### Statistical analysis

SPSS software was used for statistical analysis. Data were presented as mean ± standard deviation (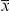
±SD); one-way ANOVA was used for intergroup comparison; differences with P<0.05 were considered statistically significant.
